# The Hair Cell α9α10 Nicotinic Acetylcholine Receptor: Odd Cousin in an Old Family

**DOI:** 10.3389/fncel.2021.785265

**Published:** 2021-11-15

**Authors:** Marcela Lipovsek, Irina Marcovich, Ana Belén Elgoyhen

**Affiliations:** ^1^Ear Institute, Faculty of Brain Sciences, University College London, London, United Kingdom; ^2^Departments of Otolaryngology & Neurology, Boston Children’s Hospital, Harvard Medical School, Boston, MA, United States; ^3^Instituto de Investigaciones en Ingeniería Genética y Biología Molecular “Dr. Héctor N. Torres” (INGEBI), Consejo Nacional de Investigaciones Científicas y Técnicas (CONICET), Buenos Aires, Argentina

**Keywords:** nicotinic acetylcholine receptors, evolution, hair cells, efferent system, ion channel

## Abstract

Nicotinic acetylcholine receptors (nAChRs) are a subfamily of pentameric ligand-gated ion channels with members identified in most eumetazoan clades. In vertebrates, they are divided into three subgroups, according to their main tissue of expression: neuronal, muscle and hair cell nAChRs. Each receptor subtype is composed of different subunits, encoded by paralogous genes. The latest to be identified are the α9 and α10 subunits, expressed in the mechanosensory hair cells of the inner ear and the lateral line, where they mediate efferent modulation. α9α10 nAChRs are the most divergent amongst all nicotinic receptors, showing marked differences in their degree of sequence conservation, their expression pattern, their subunit co-assembly rules and, most importantly, their functional properties. Here, we review recent advances in the understanding of the structure and evolution of nAChRs. We discuss the functional consequences of sequence divergence and conservation, with special emphasis on the hair cell α9α10 receptor, a seemingly distant cousin of neuronal and muscle nicotinic receptors. Finally, we highlight potential links between the evolution of the octavolateral system and the extreme divergence of vertebrate α9α10 receptors.

## Introduction

Ion channels play a myriad of functions in all domains of life. The different ion channels have evolved over billions of years, rendering an astounding spectrum of families with a wide number of members. Within the ion channels gated by the binding of ligands, the superfamily of pentameric ligand-gated ion channels (pLGICs) is the largest and most functionally diverse (Corringer et al., 2012; Jaiteh et al., 2016). pLGICs are ubiquitous in the major taxonomic groups, except multicellular plants and fungi (Jaiteh et al., 2016). The more recent discovery of pLGICs in bacterial species and Archaea has shown a striking conservation of many structural features within the entire family, even between distant prokaryotic and eukaryotic members (Tasneem et al., 2005; Bocquet et al., 2007), indicating an ancient origin for this receptor family (Jaiteh et al., 2016). The functional roles of pLGICs have been more thoroughly described in animals with bilateral symmetry (Bilateria), where they mediate fast synaptic transmission in the nervous system. In vertebrates, pLGICs are represented by the Cys-loop family and include the nicotinic acetylcholine receptors (nAChRs), serotonin type 3 receptors (5-HT_3_), gamma aminobutyric acid type A receptors (GABA_A_) and glycine receptors (Karlin and Akabas, 1995; Corringer et al., 2012; Jaiteh et al., 2016).

## Pentameric Ligand-Gated Ion Channels: Structure and Evolution

**Figure 1:**
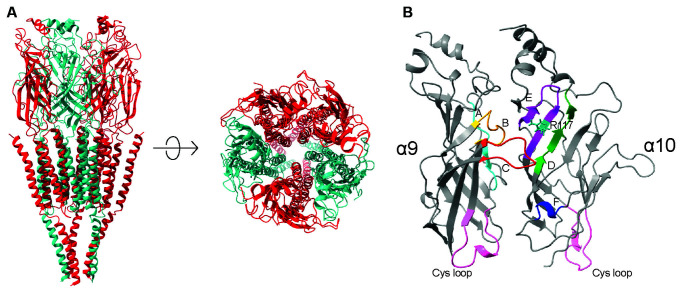
**(A)** Ribbon structure of a pentameric nicotinic acetylcholine receptor, showing the arrangement of subunits around the channel pore. **(B)** Detailed view of the ligand binding site of an α9α10 nAChRs receptor. The locations of the conserved loops that contribute to the binding site are highlighted in colour. The Cys-loops are highlighted in pink.

Pentameric ligand-gated ion channels of eukaryotes and prokaryotes exhibit relatively low amino acid sequence identity (18%–20%), but they share key common structural features. Receptor subunits have a similar domain organization and transmembrane topology, with motifs that are conserved in the entire family across species and are necessary for receptor function (Tasneem et al., 2005; Bocquet et al., 2007; Corringer et al., 2012; Jaiteh et al., 2016). Numerous 3D crystallographic or electron microscopy resolution structures of both prokaryotic (Hilf and Dutzler, 2008, 2009; Bocquet et al., 2009) and eukaryotic (Unwin, 1995, 2005; Miyazawa et al., 2003; Hibbs and Gouaux, 2011; Althoff et al., 2014; Hassaine et al., 2014; Miller and Aricescu, 2014; Du et al., 2015; Morales-Perez et al., 2016) pLGICs show a similar fivefold symmetrical arrangement of subunits around a central pore, with defined extracellular and transmembrane (TM) domains (Figure 1A). The extracellular domain folds into a highly conserved immunoglobulin-like β-sandwich (which includes 10 β-sheets) and contains the orthosteric ligand binding sites. The TM domain consists of four α-helices, with TM2 lining the channel pore, surrounded by a ring made of TM1 and TM3 α-helices. TM4 is the most peripheral helix and it interacts with the membrane lipid bilayer (Karlin, 2002; Corringer et al., 2012). The conserved 3D structure of all pLGICs supports a common phylogenetic origin for the evolutionary distant eukaryote and prokaryote pentameric receptors. Moreover, a proline residue in the extracellular domain at the loop connecting the β6 and β7 strands has been under strong selective pressure, is conserved in all pLGICs and is the basis for the proposal to name this family as the “Pro-Loop” receptors (Jaiteh et al., 2016).

Pentameric ligand-gated ion channels are ubiquitous in the major taxonomic groups except multicellular plants and fungi. Although pLGICs were first identified in vertebrates (Noda et al., 1982) and described as Cys-loop receptors due to the presence of a cysteine disulfide bridge that stabilizes the β6–β7 loop in the N-terminal extracellular domain, the discovery of Cys-less members indicates that this is not a plesiomorphic characteristic of the entire superfamily (Jaiteh et al., 2016). The residues most prominently conserved across the entire superfamily, including the proline residue mentioned above, participate in the interactions between the extracellular and TM domains crucial for signal transduction during channel opening (Jaiteh et al., 2016).

Cys-less pLGICs are present in Bacteria and Archaea and unicellular eukaryotes. Among Eumetazoans, Cys-less pLGICs have been identified in cnidarians (e.g., polyps and jellyfish), echinoderms (e.g., starfish and sea urchins), nematodes (e.g., roundworms), platyhelminths (e.g., flatworms), annelids (e.g., earthworms), and molluscs (e.g., snails, octopus and clams). They are absent from vertebrates and only found in a cephalochordate (e.g., lancelet) and a tunicate (e.g., sea squirts; Jaiteh et al., 2016).

Evolutionary analysis suggests that Cys-loop pLGICs are only present in eukaryotes and form a monophyletic group, originating from a single ancestor gene. This later diverged, generating the extant complement of subunits, which includes a subdivision into anionic and cationic receptors, that predates the origin of metazoans (Jaiteh et al., 2016).

## Cys-Loop Receptors

In Bilateria, pLGICs are most prominently represented by the family of Cys-loop receptors, with all member subunits containing a disulfide cysteine bridge which closes a β6-β7 loop comprising 13 amino acids (Changeux et al., 1987; Maricq et al., 1991; Corringer et al., 2012; Smart and Stephenson, 2019). Based on mutagenesis studies, this cysteine bridge has been proposed to be essential for the correct folding and assembly of Cys-loop receptors (Mishina et al., 1985; Blount and Merlie, 1990; Rajendra et al., 1995). Vertebrate Cys-loop receptors are divided into two subfamilies: the cationic nAChRs and 5-HT_3_ receptors and the anionic GABA_A_ and glycine receptors, which serve excitatory and inhibitory synaptic transmission in the nervous system, respectively (Nemecz et al., 2016). The Cys-loop family also includes zinc-gated channels that are absent in some mammalian species, and whose function is still not well understood (Davies et al., 2003). Both nAChRs and GABA_A_ receptors are composed of a wide array of subunits, leading to a wide range of possible pentameric combinatorial assemblies. On the other hand, vertebrate genomes contain genes coding for only five subunits of 5-HT_3_ receptors and five subunits of glycine receptors, leading to a more limited number of possible combinatorial assemblies (Nemecz et al., 2016).

In serotonin-gated Cys-loop receptors, 5-HT_3_A is the only subunit that assembles into homomeric receptors. Functional diversity is achieved by the co-assembly of 5-HT_3_A with either 5-HT_3_B, C, D, or E in heteromeric combinations (Niesler et al., 2007; Barnes et al., 2009; Holbrook et al., 2009). Moreover, diversity is further increased by alternative splicing of the genes encoding the 5-HTR_3_A and E subunits (Brüss et al., 2000; Niesler, 2011). Functional glycine receptors are either homopentamers of α subunits, or heteropentamers composed of two α and three β subunits (Betz and Laube, 2006). Four α subunits (α1-α4) and one β subunit have been identified. Further diversity arises from the alternative splicing of α1 (α1^INS^ and α1^del^), α2 (α2A and α2B), α3 (α3S and α3L) and β (βΔ7) subunits and by mRNA editing of the α2 and α3 subunits (Meier et al., 2005; Betz and Laube, 2006; Oertel et al., 2007).

GABA is the main inhibitory neurotransmitter in the mammalian nervous system, where 19 GABA_A_ receptor subunits have been identified (Simon et al., 2004; Amundarain et al., 2019; Smart and Stephenson, 2019). The subunits are divided into classes (α, β, γ, ρ, θ, ε, π, and δ) and subclasses (α1–6, β1–3, γ1–3, and ρ1–3), based on sequence identity. The diversity of subunits is further increased by alternative splicing, to which 9 out of 19 subunits are subject to, and is proposed to regulate subunit expression (Whiting et al., 1990; Simon et al., 2004). The amino acid sequence identity between subunits of the same class ranges between 70 and 80% and falls to 30–40% for subunits of different classes (Macdonald and Olsen, 1994). Individual subunits exhibit distinct but overlapping and often widespread expression patterns throughout the nervous system (Pirker et al., 2000), resulting in a large variety of GABA_A_ receptor subtypes in the brain. Native GABA_A_ receptors are mainly composed of αβγ subunits usually in a stoichiometry of 2:2:1 with identical (but not always) α and β subunits (Olsen and Sieghart, 2008; Sarto-Jackson and Sieghart, 2008; Amundarain et al., 2019; Smart and Stephenson, 2019). However, other stoichiometries can be observed. Thus, ε, π and δ subunits can replace the γ subunit, and a θ subunit can replace a β subunit. The ρ1/2/3 subunits usually assemble in homopentamers or heteropentamers (Cutting et al., 1991; Enz and Cutting, 1998). However, the assembly of ρ subunits with α1 and/or γ2 subunits has been also identified (Milligan et al., 2004; Harvey et al., 2006). Although initially described as retinal subunits, the ρ subunits are also expressed in the brain (Milligan et al., 2004; Harvey et al., 2006).

Neurons co-express multiple GABA_A_ subunits and a single neuron can express several different receptor subtypes (Olsen and Sieghart, 2008; Sarto-Jackson and Sieghart, 2008; Smart and Stephenson, 2019; Sallard et al., 2021). The most abundant subtype in the mammalian nervous system is α1β2γ2 (Rudolph and Knoflach, 2011). Currently, eleven native GABA_A_ receptors have been conclusively identified: α1β2γ2, α1βγ2, α3βγ2, α4βγ2, α4β2*δ*, α4β3*δ*, α5βγ2, α6βγ2, α6β2*δ*, α6β3*δ*, and *ρ*. Further combinations are classified with a high probability of assembly and the number of described native GABA_A_ receptors continues to increase (Olsen and Sieghart, 2008). The incorporation of different subunits to the pentamer determines the trafficking, cell surface expression, internalisation, and function of GABA_A_ receptors (Jacob et al., 2008). For example, receptors that include the γ2 subunit (except when associated with α5) cluster at the postsynaptic membrane and distribute dynamically between synaptic and extrasynaptic locations, whereas those incorporating the δ subunit appear to be exclusively extrasynaptic (Jacob et al., 2008).

## Nicotinic Acetylcholine Receptors

The nAChRs are a major branch of the Cys-loop family of the pLGIC superfamily (Corringer et al., 2012). Vertebrate nAChRs are non-selective cation channels. To date, 19 different nAChR subunits have been described in most of the main vertebrate clades: α1–α10, β1–β4, γ, δ, and ε (Karlin, 2002; Corringer et al., 2012), with α11 and β5 subunits only identified in some fish species (Pedersen et al., 2019). The nAChR subunits were initially classified into muscle and neuronal subtypes, based on their expression pattern and function, either at the neuromuscular junction or the nervous system (Karlin, 2002; Changeux et al., 1987; Le Novere and Changeux, 1995). However, this dichotomic classification required revisiting with the discovery of the hair cell nAChR subunits.

Functional nAChRs result from the assembly of either five identical or different subunits, giving rise to homomeric or heteromeric pentamers, respectively (Karlin, 2002). With the exception of α9 homomeric and α9α10 heteromeric receptors (Elgoyhen et al., 1994, 2001), all nAChR known to date respond to nicotine, thus naming the subfamily. The ligand binding site is at the interface of the extracellular domains of adjacent subunits and is formed by six structurally conserved loops (Figure 1B). Each binding site is composed of a principal component or (+) face provided by one subunit, which contributes three loops of highly conserved residues (loops A–C), and a complementary component, or (-) face, of the adjacent subunit, which contributes the reminder less conserved loops (loops D–F; Brejc et al., 2001; Unwin, 2005; Dellisanti et al., 2007). Consequently, the components of the extracellular inter-subunit binding sites are non-equivalent and their loops contribute differentially to receptor function (Karlin, 2002).

The rules that govern the combinatorial assembly of functional nAChRs are for the most part unknown, especially in the case of neuronal receptors. These are formed by diverse combinations of α2-α7 (α8 in non-mammals) and β2–4 subunits, giving rise to an extensive range of as yet not fully characterised combinatorial arrangements (Gotti et al., 2009; Zoli et al., 2015, 2018). This complexity is further extended by the combinatorial assembly of heteromeric neuronal receptors formed by the same subunits, but with alternative stoichiometry (Nelson et al., 2003; Moroni and Bermudez, 2006; Moroni et al., 2006; Krashia et al., 2010; Benallegue et al., 2013; Mazzaferro et al., 2017). In contrast, muscle receptors show a more constrained co-assembly spectrum, since they are formed by α1_2_ β1γ, and δ, or, ε and do not co-assemble with non-muscle subunits (Mishina et al., 1986; Cetin et al., 2020). Finally, nAChR subunits expressed in cochlear and vestibular hair cells have a very strict co-assembly pattern, only comprised of α9 and α10 subunits (Elgoyhen et al., 1994, 2001; Sgard et al., 2002). While α9 subunits can assemble into functional homomeric receptors when expressed in heterologous systems (Elgoyhen et al., 1994), these do not play a major role in inner ear hair cells *in vivo*, as described in mice lacking the *CHRNA10* gene, coding for the α10 subunit (Vetter et al., 2007). More importantly, neither α9 nor α10 subunits co-assemble with other nAChR subunits to form functional receptors (Elgoyhen et al., 1994; Scheffer et al., 2007). Therefore, the α9 and α10 subunits are functionally isolated from the remainder nicotinic subunits. Resulting from their expression pattern (Elgoyhen et al., 1994; Morley et al., 1998, 2018; Atlas, 2013), their distinct evolutionary trajectory when compared to other nAChRs (Franchini and Elgoyhen, 2006; Lipovsek et al., 2012; Marcovich et al., 2020), their lack of assembly with other nAChR subunits (Elgoyhen et al., 1994; Scheffer et al., 2007), and their peculiar pharmacological and biophysical properties (Elgoyhen et al., 1994, 2001; Rothlin et al., 1999, 2003; Verbitsky et al., 2000; Sgard et al., 2002; Gomez-Casati et al., 2005; Plazas et al., 2007), α9 and α10 form a separate branch within the subfamily of nAChRs. Thus, although initially included within the neuronal subgroup of nAChR subunits (Karlin, 2002), α9 and α10 subunits do not fully share functional, expression, pharmacological and evolutionary properties with neuronal subunits, and are therefore clearly non-neuronal. We propose to re-classify them as “hair cell” nAChR subunits, based on their known function mediating efferent olivocochlear inhibition of inner ear hair cells (Katz et al., 2004; Ballestero et al., 2011; Elgoyhen and Katz, 2012; Katz and Elgoyhen, 2014).

A consequence of the differences in co-assembly rules between the three subgroups of nAChRs is that muscle cells mainly express two receptor variants (one adult and one embryonic; Mishina et al., 1986), cochlear hair cells only one (Elgoyhen et al., 1994, 2001; Morley et al., 1998; Vetter et al., 1999, 2007; Gomez-Casati et al., 2005), while neurons are capable of expressing a great diversity of nAChRs (Zoli et al., 2015, 2018; Marcovich et al., 2020). For example, α7* (*, subunit containing) and α4β2* are the two most abundant nAChRs in the central nervous system (Zoli et al., 2015, 2018), whereas receptors containing α3 and β4 subunits mediate fast synaptic transmission at the autonomic ganglia (Covernton et al., 1994; Skok, 2002) and α6β2* receptors are localised presynaptically in both visual and mesostriatal pathways (Gotti et al., 2009). Moreover, neuronal receptors can contain more than two different subunits. For example, α4β2* nAChRs in some brain regions also contain the α5 subunit (Brown et al., 2007). For a comprehensive list of experimentally validated nAChR assemblies see Supplementary Table S6 in Marcovich et al. (2020).

A recent systematic gene expression re-analysis of 10 single-cell transcriptomic studies extended to the single cell level the identification of the variety of possible neuronal nAChRs assemblies (Marcovich et al., 2020). This study explored the potential spectrum of nAChRs in any given neuron with diverse neurochemical identities in different regions of the mouse nervous system. In doing so it outlined the potential complement of pentameric receptors present in each cell type, by identifying the subunit combinations that are present within a 10-fold, 100-fold or 1,000-fold range of expression level or altogether absent. As expected, neurons express a potentially wide range of neuronal nAChR variants.

The possibility of toggling between nAChR subunits incorporated into different pentameric assemblies gives rise to receptors with a wide variety of functional properties. Thus, neurons have the potential to express nAChRs with diverging ACh sensitivity, kinetics, conductance and relative cation permeability (Patrick et al., 1993; Dani and Bertrand, 2007; Zoli et al., 2015, 2018) and consequently adjust their properties to serve differential functions in different regions of the nervous system. For example, receptors composed solely of α7 subunits have low affinity for ACh, fast desensitisation kinetics and high relative calcium permeability (Cooper et al., 1991; Séguéla et al., 1993). Receptors composed of α4 and β2 subunits have a higher affinity for ACh, slower desensitisation kinetics, and lower relative calcium permeability (Cooper et al., 1991; Fucile et al., 2006; Dani and Bertrand, 2007). The inclusion of additional subunits into the pentamer also contributes to functional diversity. For instance, the incorporation of the α5 subunit increases the calcium permeability, ACh sensitivity, and desensitization kinetics of α4β2* receptors (Tapia et al., 2007; Kuryatov et al., 2008; Sciaccaluga et al., 2015). On the other hand, the incorporation of the β3 subunit to α4β2* receptors increases ACh sensitivity, without significantly affecting calcium permeability (Tapia et al., 2007; Kuryatov et al., 2008). Finally, alternative stoichiometries of the same subunit assemblies (e.g., α4_2_β2_3_ or α4_3_β2_2_) result in receptors with different sensitivity to ACh, unitary current amplitude, desensitization rate, calcium permeability, and selectivity for agonists and antagonists (Nelson et al., 2003; Moroni and Bermudez, 2006; Moroni et al., 2006; Tapia et al., 2007; Krashia et al., 2010; Mazzaferro et al., 2011, 2014, 2017; Benallegue et al., 2013; New et al., 2018), further increasing the extent of functional diversity of neuronal nAChRs.

The analysis of single-cell transcriptomic data revealed that the expression pattern of nicotinic subunits could indeed contribute to functional diversity. For example, cortical neurons that project to both the ventral posteromedial nucleus (VPM) and the posteromedial complex of the thalamus express significantly higher levels of the α5 subunit than neurons only projecting to the VPM, suggesting that the latter could have a lower density of α4α5β2* compared to α4β2* nAChRs. This might relate to the known differences in excitability of layer VI neurons (Landisman and Connors, 2007). In addition, differential co-expression patterns of nAChR subunits are also observed between four different subtypes of dopaminergic neurons in the midbrain ventral tegmental area (VTA). β2 and β3 subunits are expressed at comparable levels in all four VTA dopaminergic neuron subtypes, lower levels of α4 are present in VTA2 and VTA4, and α5 is absent in VTA3 neurons and expressed at different, but low levels in VTA1, VTA2, and VTA4. These observations suggest that the four subtypes of dopaminergic neurons might contain different levels of α4β2*, α4α5β2*, and α4β2β3* receptors. Thus, the differential modulatory control of dopaminergic neuron firing patterns exerted by cholinergic input (Maskos et al., 2005; Mameli-Engvall et al., 2006), might relate to the expression of functionally different neuronal nAChRs.

**Figure 2:**
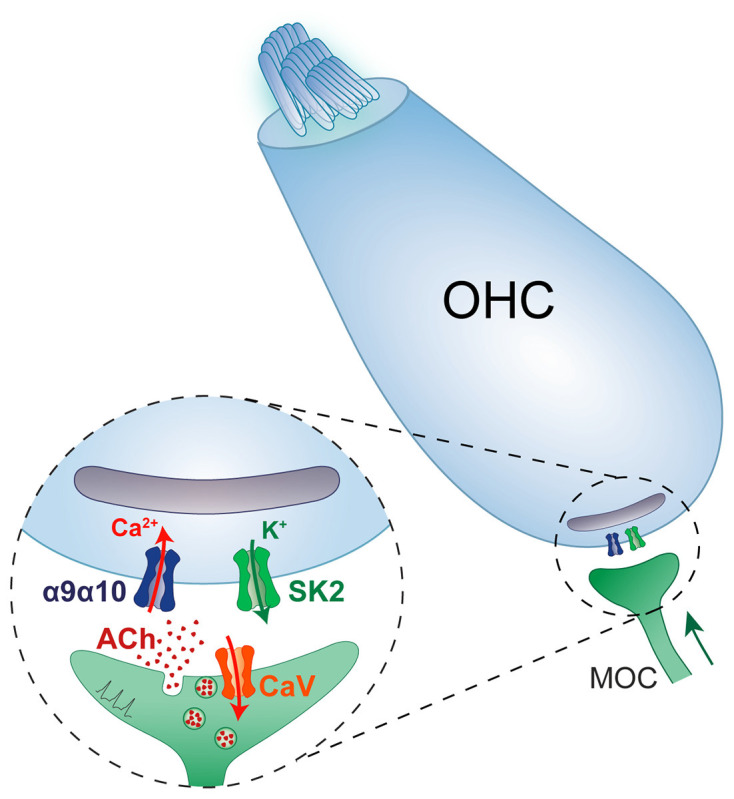
Schematic diagram of the efferent synapse between a medial olivocochlear fibre and an outer hair cell, highlighting the different channel components involved in the ACh-triggered hyperpolarisation of hair cells.

In contrast to the wide variety of neuronal nAChRs expressed in the nervous system, cochlear and vestibular hair cells only express functional α9α10 nAChRs (Elgoyhen et al., 1994, 2001; Hiel et al., 1996; Morley et al., 1998; Vetter et al., 1999, 2007; Morley and Simmons, 2002; Sgard et al., 2002). Although several studies have shown expression of α1, β2, β4, and γ transcripts (Scheffer et al., 2007; Burns et al., 2015; Cai et al., 2015; Roux et al., 2016; McInturff et al., 2018; Marcovich et al., 2020), no response to nicotine is observed in hair cells (Housley and Ashmore, 1991; Fuchs and Murrow, 1992b; Gomez-Casati et al., 2005; Ballestero et al., 2011), indicating that functional muscle or neuronal nAChRs are not present at the plasma membrane and that the mRNAs detected in hair cells most likely derive from redundant or residual transcription regulation mechanisms. In addition, acetylcholine responses are absent in α9 and α10 knockout mice (Vetter et al., 2007), indicating that only α9α10 nAChRs drive cholinergic responses in hair cells and that the lack of *CHRNA9* and/or *CHRNA10* transcription is not compensated by the expression of either muscle or neuronal nAChR genes. A striking and unique feature of the α9α10 nAChR is that, in contrast to all other nAChRs which serve excitatory neurotransmission, it elicits synaptic inhibition of hair cells. This is brought about by the secondary activation of a nearby calcium-dependent SK2 potassium channel which leads to hair cell hyperpolarization (Housley and Ashmore, 1991; Fuchs and Murrow, 1992a; Blanchet et al., 1996; Glowatzki and Fuchs, 2000; Katz et al., 2004; Gomez-Casati et al., 2005; Ballestero et al., 2011; Figure 2).

All components of the cholinergic system, including synthesis and degradation of ACh, have been identified in mammalian non-neuronal cells, including epithelial, endothelial and immune cells (Wessler et al., 1998). Moreover, alteration of the expression of nAChRs has been implicated in autoimmune and inflammatory diseases (Wang et al., 2003; Liu et al., 2017). Nicotinic subunits (such as α7, α9 and α10) and muscarinic ACh receptors, are expressed in peripheral, non-neuronal tissues, including the skin and the immune system where they play an important immunomodulatory role (Arredondo et al., 2002; Wang et al., 2003; Nguyen et al., 2004; Peng et al., 2004; Wessler and Kirkpatrick, 2008; Rosas-Ballina et al., 2011; St-Pierre et al., 2016; Fujii et al., 2017; Liu et al., 2017, 2019; Zakrzewicz et al., 2017; Zhang et al., 2020). Whether nAChRs expressed in non-neuronal tissues signal through channel activation or through alternative metabotropic pathways is still an open question (Valbuena and Lerma, 2016). In any event, during the course of evolution, the autocrine/paracrine effect of ACh could have been served by a multiple and redundant battery of expressed muscarinic and nAChRs. Overall, a potential role for peripheral function as a player in the evolutionary processes that shaped the coding sequence and expression patterns of nicotinic receptors is yet to be explored.

## Evolution of nAChRs

Numerous phylogenetic analyses of the subfamily of nAChR subunits have been performed (Ortells and Lunt, 1995; Le Novere and Changeux, 1995; Tsunoyama and Gojobori, 1998; Dent, 2006; Franchini and Elgoyhen, 2006; Lipovsek et al., 2012; Li et al., 2016; Faltine-Gonzalez and Layden, 2019; Jiao et al., 2019; Pedersen et al., 2019; Marcovich et al., 2020; Jones et al., 2021). Some of them date to the pre-genomic era, including a small number of coding sequences and leading to less informative iterations of phylogenetic tree topologies. In addition, the long evolutionary distances make the ancestral subunits, from which the entire family of extant nAChR subunits radiated, difficult to track. One of the first comprehensive analyses of bilaterian pLGIC evolution suggested that the last common ancestor to Bilateria most likely had at least an α7-like subunit, an α9-like subunit, a neuronal/muscle α-like subunit and a neuronal/muscle non α-like subunit (Dent, 2006). Overall, nAChRs are only found in Eumetazoans, and the addition of new data from cnidarian genomes showed the independent radiation of nAChR genes in the cnidarian and bilaterian lineages (Faltine-Gonzalez and Layden, 2019; Jiao et al., 2019).

Several difficulties are presented when attempting to establish phylogenetic relationships within radiating protein coding families across long evolutionary distances, and the results obtained can vary depending on the methodologies used, the phylogenetic span and the number of sequences analysed. The presence of both a neuronal/muscle-like subunit and an α7-like subunit in the last common ancestor of Bilateria is strongly supported by the clear identification of corresponding groups of subunits in protostomes (Jones and Sattelle, 2004, 2010; Dent, 2006, 2010; Holden-Dye et al., 2013; Faltine-Gonzalez and Layden, 2019; Pedersen et al., 2019), placing the origin of these subunits before the divergence of Bilateria. In contrast, the presence of an α9-like subunit in the last common ancestor of Bilateria is less clear, only weakly supported by one report showing the grouping of annelid subunits on the same branch as the rat α9 subunit (Dent, 2006) and a separate report showing the grouping of a *C. elegans* subunit (ACR21) with the α9/α10 branch (Faltine-Gonzalez and Layden, 2019) although the latter grouping was not observed in the previous analysis performed on smaller datasets that crucially did not include sequences from cnidarian (outgroup to Bilateria) species (Dent, 2006, 2010). Therefore, though there is strong evidence that the last common ancestor of Bilateria most likely already had at least two nAChR subunits (an α7-like and a neuronal/muscle-like) the presence of a second neuronal/muscle-like and an α9-like subunits is less well supported by current data. An updated and comprehensive analysis of Eumetazoan nAChR subunits, exploiting the ever-increasing number of new genomes available, will undoubtedly shed light on this issue in the near future.

The likely scenario tracing the evolution of nAChR subunits in the vertebrate lineage is a lot clearer and has been extensively studied (Le Novere and Changeux, 1995; Ortells and Lunt, 1995; Le Novere et al., 2002; Franchini and Elgoyhen, 2006; Dent, 2010; Elgoyhen and Franchini, 2011; Lipovsek et al., 2012; Pedersen et al., 2019; Marcovich et al., 2020). Making use of sequence phylogeny, exon-intron organization, and chromosomal information for synteny analysis and identification of paralogons, Pedersen et al. (2019) have shown that the last common ancestor of vertebrates had a repertoire of 10 genes coding for nAChR subunits. They propose that the extant complement of vertebrate subunits resulted from the first and second rounds of tetraploidization which took place in the stem vertebrate branch, between 550 and 500 million years ago, duplicate gene losses and occasional *de novo* duplications and, in the teleost lineage, a third whole genome duplication. The different extant subunits are therefore encoded by paralogous genes, all proposed to derive from five paralogons (Pedersen et al., 2019).

**Figure 3:**
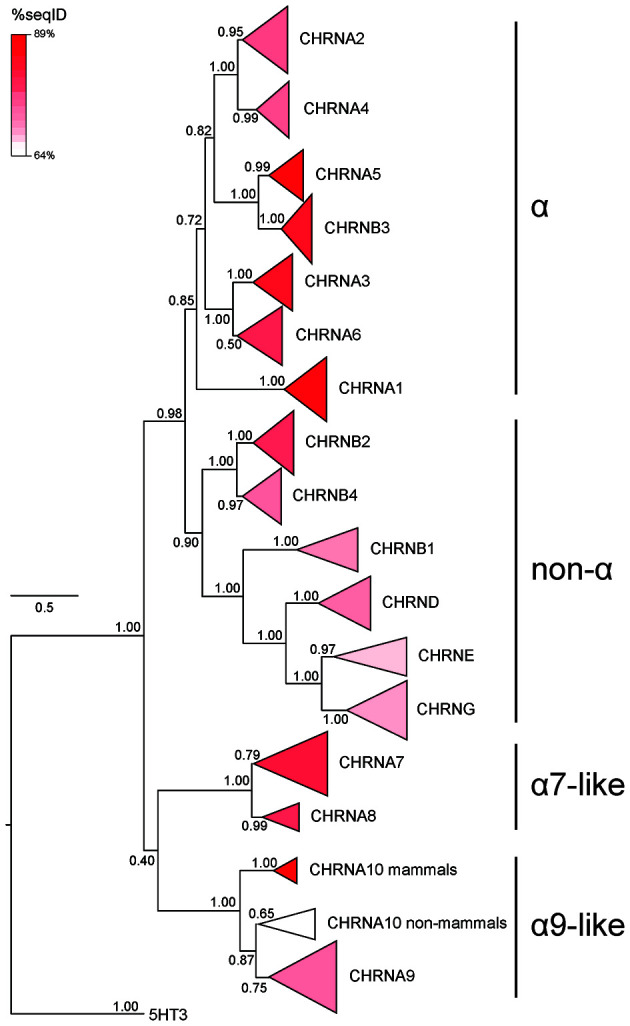
Phylogenetic tree showing the relationships between vertebrate nicotinic subunits. The branches corresponding to the same subunits of different species were collapsed to their respective node. The length of each triangle denotes sequence divergence from the corresponding node. Triangle shades depict the average sequence identity within the branch. Numbers in each branch indicate bootstrap values obtained during phylogeny construction. The scale bar indicates the number of amino acid substitutions per site. Modified from Marcovich et al. (2020), under the Creative Commons license (http://creativecommons.org/licenses/by/4.0/).

The analysis of phylogenetic trees constructed using protein coding sequences helps establish the degree of similarity and divergence between paralogous genes. Many such analyses have been performed for nAChR subunits, chiefly including vertebrate sequences. However, as mentioned above, these analyses have caveats and limitations that must be considered when drawing conclusions about the origins and relationships between members of a gene family. Overall, muscle and neuronal (excluding α7-like) subunits show a greater degree of sequence similarity amongst themselves, forming two groups comprised of α and non-α subunits. Also, α7-like subunits typically form their own branch, that may be more similar to the α9/α10 subunits (Nishino et al., 2011; Marcovich et al., 2020) or to the α/non-α group (Lipovsek et al., 2012; Pedersen et al., 2019). Finally, α9 and α10 subunits form their own group, in line with their lowest degree of sequence identity when compared against all other nAChR subunits. Of note, the position of the α9/α10 group as the outermost branch on the tree of vertebrate nAChR paralogues (although see Figure 3 and Marcovich et al., 2020) has been repeatedly used as evidence for a more ancestral origin of α9/α10 subunits, proposing that an α9-like subunit was the first one to split within the subfamily (Ortells and Lunt, 1995; Le Novere and Changeux, 1995; Tsunoyama and Gojobori, 1998; Le Novere et al., 2002). However, it must be pointed out that phylogenetic trees built based on coding sequence alignments merely reflect the degree of sequence identity. Separate branches and/or greater distance metrics could either result from: (1) the slow accumulation of changes through longer evolutionary times; (2) the rapid accumulation of sequence changes along shorter evolutionary times; or (3) a combination of both. The observation of higher-than-expected rates of non-synonymous substitutions (Franchini and Elgoyhen, 2006; Lipovsek et al., 2014; Pedersen et al., 2019), strong signals of positive selection (Franchini and Elgoyhen, 2006; Marcovich et al., 2020) and higher rates of site-specific evolutionary shifts in the amino acid biochemical state in the genes coding for α9 and α10 subunits (Marcovich et al., 2020) support the second scenario. In summary, although the α9 and α10 subunits are categorically the most divergent amongst the repertoire of vertebrate nAChR subunits, more evidence is required to establish whether they were the first branch to split from the ancestral nicotinic subunit of Bilateria (or Metazoa). As discussed above, this scenario is not currently supported, due to the lack of unequivocally identified α9-like subunits in protostomes. Once again, further analysis of newly available genomes covering all branches of Bilateria and, more importantly, outgroups such us Cnidarians, will contribute to addressing this question. Moreover, coding sequence-based analysis can be complemented by studying other aspects of gene and genome architecture (e.g., intron-exon structure, synteny, promoter/enhancer sequences), alongside analysis on the conservation and divergence of protein assembly and functional properties.

Finally, with the exception of the loss of the α7-like α8 subunit in mammals (Dent, 2006, 2010; Marcovich et al., 2020), the same repertoire of paralogous genes coding for nAChR subunits has been retained across the branch of tetrapod vertebrates. This high level of subunit conservation suggests a family-wide negative selection pressure for the loss of paralog genes. Moreover, it points towards an important functional relevance of each subunit across the clade, that may have greatly influenced their evolutionary history.

## Evolution of Neuronal nAChRs

In tetrapods, there are 10 neuronal nAChR subunits [α2–α8 (α2–α7 in mammals) and β2–β4], resulting in a plethora of subunit co-expression patterns and co-assembly possibilities (Marcovich et al., 2020). Due to the widespread expression of neuronal subunits in different regions of the nervous system and in different neuronal types within a region (Marcovich et al., 2020), randomly acquired coding sequence mutations that lead to changes in functional properties may have had deleterious effects on alternative receptor combinations expressed in different neuronal cell types. Therefore, the coding sequences of neuronal nAChRs were under strong negative selection pressure (Franchini and Elgoyhen, 2006; Elgoyhen and Franchini, 2011; Lipovsek et al., 2012). Moreover, an analysis of site-specific evolutionary shifts in amino acid biochemical state failed to identify between-clade functional divergence at the sequence level on neuronal subunits (Marcovich et al., 2020). This is mirrored by the observation that the biophysical and pharmacological properties of the two most abundant neuronal nAChRs (α4β2 and α7 receptors) in three tetrapod species (rat, chicken, and frog) show a high degree of conservation, with the inferred character state for the functional properties of the amniote and tetrapod ancestral receptors almost mirroring those of their extant counterparts (Marcovich et al., 2020).

On the basis of all the above, Marcovich et al. (2020) have proposed that in neurons, functional diversification could have arisen from stochastic changes in the expression patterns of receptor subunits, resulting in a given cell changing the subtype of receptor it expresses, while preserving individual subunit functionality. Consequently, the selection pressure for functionally distinct neuronal nAChRs could have more likely fallen on stochastic changes that affect the expression patterns of alternative subunits. Moreover, an additional substrate for functional divergence of neuronal nAChRs may derive from changes in the expression pattern and/ or function of chaperon proteins that influence the post-translational assembly and surface expression of neuronal subunits (Koperniak et al., 2013; Gu et al., 2016, 2019; Matta et al., 2017; Dawe et al., 2019; Kweon et al., 2020). This is overall in agreement with the low degree of coding sequence divergence observed for most brain expressed genes, associated with random changes in their non-coding regions leading to differential expression patterns across brain areas or species (Hoekstra and Coyne, 2007; Haygood et al., 2010). A comprehensive analysis of the promoter and enhancer regulatory regions of the genes coding for nAChR subunits, accessory and chaperon proteins and other components of cholinergic synapses, will contribute to improving our understanding of their evolution in the vertebrate nervous system.

## Evolution of Muscle nAChRs

Muscle nAChRs are composed of two α1, one β1, and one δ subunits, together with one γ-subunit in the fetal muscle nAChR, or an ε-subunit in the adult muscle nAChR (Karlin, 2002). This switch in subunit composition results in changes in several functional properties (recently reviewed in Cetin et al., 2020). The complement of five muscle nAChR subunits is conserved in vertebrates, though some non-mammalian muscle orthologs are yet to be unequivocally mapped to their respective genomes. For example, the β1 and ε subunits have not been yet annotated on several of the current avian, reptilian, and/or amphibian genome assemblies. Of note, the β1 subunit has been identified, using more intensive search tools, in the annotated genomes of painted and Chinese soft-shell turtles, python, and American alligator. The ε subunit has been likewise reported in python, turtle, American alligator, and frog genomes (Pedersen et al., 2019; Marcovich et al., 2020). Both the β1 and ε subunits from *Xenopus* have been cloned from a cDNA library and functionally studied (Kullberg et al., 1994; Sullivan et al., 2004), and a partial cDNA clone for the β1 subunit from chicken has also been reported (Moss et al., 1987). This indicates that as genome coverage, assembly, and annotation improve for avian/reptilian species, in particular for the underrepresented micro chromosomes (e.g., International Chicken Genome Sequencing Consortium, 2004; Liu et al., 2021), the β1 and ε subunits are likely to be definitively mapped. For example, a transcript (ENSGALG00000054377.1) annotated in the latest *Gallus gallus* genome assembly (GRCg6a) and localised to an unspecified scaffold, corresponds to a reported partial cDNA clone of the chicken β1 subunit (Moss et al., 1987). Finally, a local duplication of the *CHRNB1* gene has been described in spotted gar and teleosts (Pedersen et al., 2019).

Variability in the coding sequence of muscle nAChR subunits is somewhat higher than the general conservation observed for neuronal subunits. Overall, sequence identity is lower for all vertebrate muscle subunits, with the highest divergence observed for the γ and ε subunits (Marcovich et al., 2020). Additionally, phylogenetic analysis of vertebrate subunits shows varying rates of amino acid changes among tetrapod coding sequences for the ε subunit (Pedersen et al., 2019).

The change in subunit composition from the fetal to the adult muscle nAChR conformation affects receptor expression, localisation, and functional properties (Tapia et al., 2012; Cetin et al., 2020). The switch from γ to ε subunit is accompanied by the clustering of receptors on well-defined end plates innervated by a single fibre (Cetin et al., 2020). Functionally, the fetal muscle nAChR shows lower conductance, but significantly longer opening times, accompanied by higher sensitivity to ACh and choline, slower recovery from desensitised states and lower relative calcium permeability than the adult counterpart (Fucile et al., 2006; Cetin et al., 2020). This functional shift is closely linked to the expression and morphological changes that characterise the maturation of the neuromuscular junction. For example, replacing the fetal γ subunit with a chimeric γ^ε^ subunit which bares functional ε-like properties, substantially alters the innervation pattern of muscle by motor nerve fibres, resulting in the formation of functional neuromuscular synapses outside the central end-plate band region in the diaphragm (Koenen et al., 2005).

In addition to the striking developmental differences between subtypes of muscle nAChRs driven by changes in subunit composition, interspecies differences in functional properties have also been observed. The human adult muscle receptor has significantly higher calcium permeability than the mouse counterpart, and this is driven by differences within the ε subunit (Fucile et al., 2006). The physiological consequences of the increased calcium permeability are yet to be explored. However, this observation suggests that muscle receptors may sit somewhere in between neuronal and hair cell receptors in terms of the likely targets of functional selection pressure, with both changes in subunit composition and coding sequence contributing to functional changes.

## Evolution of Hair Cell nAChRs

Hair cell α9α10 nAChRs are distinct from other nicotinic receptors in that a greater divergence in their coding sequence has translated into differential functional properties across clades (Lipovsek et al., 2012, 2014; Boffi et al., 2017; Marcovich et al., 2020; Moglie et al., 2021a). The only functional nAChR in inner ear hair cells is composed of α9 and α10 subunits. Extensive phylogenetic analysis of their coding sequences has revealed unique features about their evolutionary history (Franchini and Elgoyhen, 2006; Lipovsek et al., 2012, 2014; Marcovich et al., 2020). Most notably, while the sequences for each of the vertebrate nAChR subunits group within their own respective branches, denoting a high degree of coding sequence conservation, α10 subunits are unique in presenting a segregated grouping of orthologs, with non-mammalian α10 subunits as a sister group to all α9 subunits, and mammalian α10 subunits as an outgroup to the α9/non-mammalian α10 branch (Franchini and Elgoyhen, 2006; Lipovsek et al., 2012; Faltine-Gonzalez and Layden, 2019; Marcovich et al., 2020). The segregated grouping of α10 orthologs is due to an overall low percentage of amino acid sequence identity amongst vertebrate α10 subunits, resulting from a high rate of acquisition of non-synonymous substitutions in the coding region of mammalian α10 subunits (Franchini and Elgoyhen, 2006; Lipovsek et al., 2012), followed by high sequence conservation within the mammalian lineage (Franchini and Elgoyhen, 2006; Lipovsek et al., 2012; Marcovich et al., 2020). A faster rate in amino acid changes for the *CHRNA10* gene in humans, mouse, and opossum compared to other non-mammalian vertebrates has also been described (Pedersen et al., 2019). These non-synonymous changes observed in mammalian *CHRNA10* could have resulted from Darwinian positive selection. Indeed, signatures of positive selection acting on *CHRNA10* coding sequences have been observed utilising different molecular evolution analysis, including Ka/Ks (non-synonymous substitutions per non-synonymous site/synonymous substitutions per synonymous site) and codon-based likelihood models (Franchini and Elgoyhen, 2006; Lipovsek et al., 2012). Moreover, the search for site-specific shifts in the amino acid biochemical state between clades (Gu et al., 2013), indicates functionally significant amino acid changes when comparing α10 mammalian subunits vs. their sauropsid counterparts (Marcovich et al., 2020).

Several of the positively selected sites identified in the α10 subunit are located within the ligand-binding and gating regions of the extracellular domain (Franchini and Elgoyhen, 2006; Lipovsek et al., 2012). Moreover, the rat α10 subunit shows a relative excess of positively charged residues (R and K) in the N-terminal extracellular domain compared to chicken α10, and chicken and rat α9 subunits (Boffi et al., 2017), which could potentially affect the interactions with the ligand through electrostatic repulsion. For example, residue 117 (numbering corresponds to *Torpedo* α1 subunit mature protein (Karlin, 2002) of Loop E in the complementary component of the binding site, is a positively charged arginine (R117, Figure 1B) in mammalian α10 subunits and a non-charged threonine or methionine in α9 and non-mammalian α10 subunits. Homology models of the extracellular domain with ACh docked in the binding site show that the positively charged R117 in α10 is located ~8–9 Å from the ACh amino group. It could therefore directly interact with the ligand and contribute to the differences in ligand binding and gating observed between rat and chicken α9α10 nAChRs (Boffi et al., 2017).

Overall, when comparing functional properties, stark differences are observed between mammalian and non-mammalian α9α10 nAChRs, which may, in turn, relate to the accumulation of amino acid changes within mammalian α10 subunits. First, chicken, but not rat α10 subunits, assemble into functional homomeric receptors (Lipovsek et al., 2014; Moglie et al., 2021a). Second, while the chicken α10 subunit can contribute both principal and complementary components to the ligand binding site, the accumulation of non-synonymous substitutions in mammalian α10 subunits suggests a potentially defective contribution of rat (but not chicken) α10 subunits to complementary components of the binding site of α9α10 nAChRs. Site-directed mutagenesis experiments, ligand binding assays, and molecular docking studies provide experimental support for this hypothesis (Boffi et al., 2017).

In addition, whereas the complementary face of the α10 subunit does not play an important role in the activation of the rat α9α10 receptor by ACh (Boffi et al., 2017), it is strictly required for receptor activation by choline (Moglie et al., 2021a). Therefore, the evolutionary changes acquired in the mammalian α10 nAChR subunit resulted in the loss of choline acting as a full agonist in rat α9α10 nAChRs (Moglie et al., 2021a). Since choline is present at the synaptic cleft, due to ACh hydrolysis by acetylcholinesterase, this difference in the efficacy of choline on α9α10 nAChRs might result in different kinetics of efferent synapses across species.

Molecular evolution analysis of the α9 subunits indicated no statistically significant evidence of positive selection on the coding sequences (Franchini and Elgoyhen, 2006; Lipovsek et al., 2014). However, mammalian α9 subunits show a higher prevalence of non-synonymous substitutions (Lipovsek et al., 2014) and functionally significant amino acid changes along the protein when comparing the α9 mammalian vs. sauropsid (birds and reptiles) clades (Marcovich et al., 2020). Moreover, ancestral sequence reconstruction of all the nodes of the α9 tetrapod phylogeny indicates a greater degree of divergence from the common amniote ancestor for the mammalian lineage, contrasting against the greater sequence conservation on the sauropsid lineage (Lipovsek et al., 2014). A zoom into the alignment of the extant tetrapod sequences and those predicted for the major clade nodes showed that when comparing mammalian vs. sauropsid α9 amino acid sequences, both branching from the ancestral amniote, 42 sites had non-synonymous branch-specific substitutions and that the majority of them (36 of the 42 changes) occurred in the mammalian lineage (Lipovsek et al., 2014). Altogether, the DIVERGE analysis coupled to the ancestral sequence reconstruction, suggest that clade-specific functionally significant amino acid changes also occurred during the evolution of mammalian α9 nAChR subunits, albeit with a lower prevalence when compared to α10 subunits. Using molecular dynamics simulations and an evolutionary-based mutagenesis strategy, Lipovsek et al. (2014) identified three specific amino acid substitutions in the α9 subunit that rendered a high calcium permeable mammalian (but not chicken) α9α10 nAChR, stemming from a low calcium permeable amniote ancestor. These sites are located at the extracellular vestibule (D110 and S127) and at the exit of the channel pore (4’A; Lipovsek et al., 2014), and not at the pore-forming transmembrane region 2 of the protein as previously proposed (Galzi et al., 1992; Bertrand et al., 1993; Tapia et al., 2007). Thus, though lower in numbers, mammalian-specific non-synonymous substitutions on the α9 subunit led to important functional changes in the properties of the α9α10 nAChR.

The phylogenetic analysis of the entire nAChR family shows that the average percentage of sequence identity between all pairs of sequences is lowest for non-vertebrate α10 subunits (64.25%; Figure 3 and Marcovich et al., 2020). This suggests that the functional properties of α9α10 nAChRs may not only differ when comparing mammalian vs. non-mammalian vertebrates but also along other branches of the tetrapod tree. In order to test this hypothesis, Marcovich et al. (2020) performed a comprehensive analysis of functional and biophysical properties of α9α10 receptors, comparing three representative tetrapod species (rat, chicken, and frog), and observed striking differences across them, denoting major functional divergence. This contrasts the high degree of functional conservation observed for tetrapod neuronal (α4β2 and α7) nAChRs, as discussed above.

Taken together, the inter-clade sequence divergence and the positive selection of non-synonymous substitutions in mammalian α9 and/or α10 subunits, indicate that the evolution of the hair cell nAChR has been dominated by functionally significant changes on the coding sequences. This evolutionary trajectory of the hair cell nAChR mirrors what has been recently described for an increasing number of inner ear expressed genes. Noticeably, as much as 13% of 1,300 inner ears expressed genes show signatures of positive selection in the mammalian lineage, spotting adaptive molecular evolution as a major player in the emergence of morphological and functional innovations in the mammalian inner ear (Pisciottano et al., 2019).

## Evolution of The Octavolateral Efferent System

The main functional role described to date for the α9α10 nAChR is to mediate transmission at efferent fibres—hair cells synapses. It is therefore important to analyse the distinct evolutionary trajectory of this receptor within the context of the octavolateral system. This comprises the lateral line, vestibular and auditory sensory modalities that utilise highly specialised mechanosensory hair cells, equipped with stereocilia at their apical ends, for the detection of vibrations originating from water waves, sounds, and head and body movements. The origins of epithelial mechanosensory hair cells can be traced to the earliest vertebrates (Manley and Fuchs, 2011; Sienknecht et al., 2014; Arendt et al., 2016; Fritzsch and Elliott, 2017). Homologous mechanosensory cells have also been described in tunicates, the invertebrate chordates that are a sister group of vertebrates (Manni et al., 2006; Rigon et al., 2018).

Cholinergic responses, driven by the α9α10 receptor, have been most extensively studied in rodents (Katz et al., 2004, 2011; Gomez-Casati et al., 2005; Lipovsek et al., 2012; Katz and Elgoyhen, 2014; Moglie et al., 2018, 2021b; Kearney et al., 2019) and avian (Fuchs and Murrow, 1992a,b; Lipovsek et al., 2014; Moglie et al., 2021a) auditory hair cells. Additionally, functional hair cell nAChRs have also been reported in reptiles and fish (Art and Fettiplace, 1984; Art et al., 1985; Holt et al., 2006; Parks et al., 2017; Carpaneto Freixas et al., 2021). To date, the presence of hair cell-like nAChRs is yet to be described in non-vertebrate chordates. However, the identification of such receptors in, for example, mechanosensory cells of the ascidian coronal organ, would lend additional support to the hypothesis of a common origin for chordate mechanosensory cells.

Hair cells receive afferent innervation, through which they relay mechanosensory information to the brain, and efferent innervation, that modulates hair cell activity. Efferent innervation is a prominent feature of mechanosensory organs, observed contacting mechanosensory cells in vertebrates, in the tunicate coronal organ (Manni et al., 2006; Rigon et al., 2018), the statocyst of octopus (Colmers, 1982) and the Johnston’s organ of mosquitoes (Andrés et al., 2016). However, efferent neurons are unlikely to represent homologous cell types across Bilateria. In vertebrates, efferent innervation is prominent in the inner ear, targeting both vestibular and auditory sensory epithelia, and in lateral line neuromasts (although it may have been secondarily lost in cyclostomes; Rigon et al., 2018). It is therefore as old as, and has co-evolved with, hair cells (Manley and Köppl, 1998). The cell bodies of efferent neurons are located in the hindbrain. A single efferent nucleus is present in diapsids, although in birds and some reptiles there is a partial segregation of auditory and vestibular efferents, with the latter located more dorsally (Holt et al., 2011; Cullen and Wei, 2021). In mammals, sensory modalities are completely segregated, with vestibular efferent neurons located in the dorsal hindbrain, and auditory efferent neurons localised ventrally, within the olivary complex (Holt et al., 2011; Di Bonito and Studer, 2017). In line with this, efferent innervation has been described to produce a global control of a range of end organs, including auditory, vestibular and, if present, lateral line (Manley and Köppl, 1998; Holt et al., 2011; Köppl, 2011b; Sienknecht et al., 2014). Functional studies in fish and amphibians suggest that efferent activity works as a shut-off system to prevent desensitization of peripheral sensory systems and aids detection of external vs. self-generated stimuli (Lunsford et al., 2019; Pichler and Lagnado, 2020). In amniotes, the efferent system likely adapted to provide anti-masking effects to improve signal detection (Guinan, 2010). Based on the spatial and developmental associations of the efferent neurons with facial motor neurons, in addition to their cholinergic nature, it has been proposed that inner ear efferents are evolutionarily related to facial motor neurons (Fritzsch and Elliott, 2017; Di Bonito and Studer, 2017; Frank and Goodrich, 2018).

The segregation of vestibular and auditory efferent neurons is likely part of widespread changes in the octavolateral system that followed the transition to land and ultimately led to specialisations for the detection of airborne sounds (Manley, 2000, 2017; Fritzsch and Straka, 2014; Grothe and Pecka, 2014; Clack, 2015; Carr and Christensen-Dalsgaard, 2016). The independent emergence of at least five variants of a tympanic middle ear, more than 100 million years after the separation of the tetrapod lineages (Manley, 2000; Anthwal et al., 2013; Clack, 2015; Carr and Christensen-Dalsgaard, 2016; Tucker, 2017), was accompanied by parallel evolutionary processes in the auditory systems of amniotes, that also involved the independent elongation of the sensory epithelia, extension of the hearing range to higher frequencies, and elaboration of passive and active sound amplification mechanisms, leading to fine-tuning of sound detection (Hudspeth, 1997; Manley, 2000, 2017; Dallos, 2008). In addition, mammals and sauropsids underwent independent specialization of hair cell types, segregating, partially in birds and completely in mammals, their phonoreception [tall hair cells in birds and inner hair cells (IHCs) in mammals], and sound amplification functions [short hair cells in birds and outer hair cells (OHCs) in mammals; Köppl, 2011a]. Moreover, mammals developed a novel mechanism of active sound amplification and basilar membrane fine-tuning based on OHC length changes termed somatic electromotility (Brownell et al., 1985). In adult mammals, medial olivocochlear efferent fibres synapse directly onto OHCs and modulate somatic electromotility, while lateral olivocochlear fibres contact the afferent synaptic boutons that, in turn, contact IHCs. During a short developmental time window, medial olivocochlear fibres directly contact IHCs (Glowatzki and Fuchs, 2000; Katz et al., 2004). During this period, efferent activity modulates the spontaneous activity of IHCs, affecting the maturation of IHCs themselves (Johnson et al., 2013) and of tonotopic maps along the ascending auditory pathway (Clause et al., 2014; Di Guilmi et al., 2019).

Overall, the degree of specialisation reached by amniote, and in particular mammalian, hearing highlights the strong evolutionary pressures that shaped the different components of the auditory system. The expansion of the hearing range to higher frequencies and the emergence of an active amplification mechanism for its fine-tuning presented new challenges for their modulation. In this context, the evolutionary changes in the coding sequence of the α9α10 nAChRs likely accompanied the specialization of the efferent system across the different vertebrate clades. In particular, a number of functional properties of mammalian α9α10 nAChRs suggest a role in highly reliable, high-frequency synaptic transmission for the modulation of electromotile OHCs. Thus, exclusively in mammals, efferent activation of α9α10 nAChRs presents a different set of challenges for efferent modulation, since it inhibits OHC somatic electromotility, which is driven by the motor protein prestin (Zheng et al., 2000). Noticeably, prestin and βV giant spectrin (a major component of the OHCs’ cortical cytoskeleton) also show signals of positive selection in mammals, accompanying the acquisition of somatic electromotility (Franchini and Elgoyhen, 2006; Elgoyhen and Franchini, 2011; Cortese et al., 2017), and representing a prominent example of evolutionary processes focussed on the coding sequence of inner ear-specific genes (Pisciottano et al., 2019).

Although across species calcium influx through α9α10 nAChRs activates SK potassium channels (Fuchs and Murrow, 1992a; Glowatzki and Fuchs, 2000), as described above, mammalian α9α10 nAChRs have a higher calcium permeability than their avian counterparts (Lipovsek et al., 2012, 2014). This differential calcium permeability might accompany alternative demands in efferent modulation of hair cell activity across species. First, a strong influx of calcium may be required for the activation of large conductance, voltage, and low-calcium-sensitive BK potassium channels that mediate hyperpolarization of OHCs in the basal higher frequency regions of the cochlea (Wersinger et al., 2010; Rohmann et al., 2015). Second, in addition to activating SK (or BK) potassium channels, calcium entry through α9α10 nAChRs is also involved in triggering calcium-induced calcium-release from postsynaptic cisterns that are present at efferent synapsis of birds and mammals (Fuchs et al., 2014; Im et al., 2014; Fuchs and Lauer, 2019). However, the relative contributions of calcium influx and store release differs between avian and mammalian hair cells, with chicken short hair cells likely relying more heavily on the latter (Lipovsek et al., 2012). Third, it can be hypothesized that in mammals the time course of potassium channel activation (and therefore OHC inhibition) would rely mainly on calcium entry through α9α10 receptors, and therefore more precisely follow efferent fibre activity allowing for a graded modulation of the cochlear amplifier, as opposed to the all-or-none feature typical of calcium release from intracellular stores. Finally, while in adult mammalian hair cells efferent fibres directly contact OHCs and not the IHCs that release glutamate onto afferent auditory fibres, efferent innervation in birds and amphibians coexists with glutamate releasing afferent innervation in the same sensory hair cells (Simmons et al., 1996), with the exception of the extremely tall and short hair cells in some birds. Therefore, in these non-mammalian clades, limiting the efferent-mediated calcium influx may be fundamental to avoid a possible efferent-to-afferent calcium spillover (Moglie et al., 2018; Moglie et al., 2021b), which could lead to glutamate release independent of sound driven mechanosensory activation. The low calcium permeability of chicken α9α10 nAChRs (Lipovsek et al., 2012, 2014) and the very high desensitization kinetics of amphibian α9α10 receptors (Marcovich et al., 2020) may therefore be crucial to limit a potential hair cell calcium load triggered by efferent activity.

Another functional consequence of the accumulated changes in the amino acid sequences of the α9α10 receptor is the differential efficacy of choline for receptor activation. While choline elicits maximal responses in recombinant and native chicken α9α10 receptors, it behaves as a weak partial agonist (and competitive antagonist) of rodent α9α10 receptors (Moglie et al., 2021a). Since choline is the metabolite produced by ACh degradation at the synaptic cleft, its interaction with the α9α10 nAChR might influence the kinetics of synaptic transmission. Thus, in chicken, choline will continue to activate the receptor until it is removed from the synaptic cleft, resulting in longer post-synaptic responses subjected to large variations and poor temporal tuning. In contrast, in mammals, the termination of α9α10 responses would be determined by the fast kinetics of acetylcholinesterase activity (Hall, 1973) and the degradation of ACh to choline would limit the time-course and improve the reliability of synaptic responses. This may be fundamental for the modulation of the prestin-driven amplifier of mammalian OHCs since it might allow the characteristic post-synaptic summation of efferent responses that faithfully reproduce the high frequency activity of efferent medial olivocochlear fibres (Ballestero et al., 2011).

## Discussion

The hair cell α9α10 receptor is an unusual nAChR. It differs from its muscle and neuronal cousins across many features. At the coding sequence level, it shows the greatest degree of divergence of all vertebrate nicotinic receptors, with clear signs of positive selection and functional shifts in amino acid residues. At the expression level, it shows restricted expression patterns, with co-expression of both α9 and α10 limited almost exclusively to inner ear or lateral line hair cells, the only cell type where functional α9α10 receptors have been identified to date. At the subunit assembly level, α9 and α10 show remarkable isolation, only forming functional pentamers with each other, and are likely to require completely different sets of chaperone and accessory proteins. Finally, it is at the functional level where α9α10 receptors most clearly show their colours, in stark contrast to other nAChRs, with numerous differences between α9α10 receptors across vertebrate clades. All these have delineated (or has been influenced by) a unique evolutionary trajectory for the hair cell receptor along the vertebrate phylogeny, that contrasts that of other members of the family. Moreover, amounting evidence supports a close link between the evolutionary processes affecting the hair cell receptor and those that have shaped the octavolateral system.

Ever since the cloning and functional characterisation of the first α9 subunit (Elgoyhen et al., 1994), the peculiarities of the hair cell receptor have made it an interesting, yet challenging area of research. Overall, the study of α9α10 receptors has contributed to deepening our knowledge of nicotinic acetylcholine receptors. Comparative studies across the gene family have led to the formulation of new hypotheses about the evolutionary processes that shaped its members (Marcovich et al., 2020). A corollary of the divergent evolutionary history of the α9α10 nAChR has been the suggestion that the hair cell receptor is the most ancestral member of the group of paralogue genes. Although there is a clear consensus that the α9 and α10 subunits were indeed present in the last common ancestor of all vertebrates, alongside most other nicotinic subunits, no evidence to date supports the notion that α9-like subunits represent neither the ancestral state of nicotinic subunits nor that they were the first ones to branch-off from the original stem branch of nicotinic subunits. As discussed above, the presence of an α9-like subunit in the last common ancestor to Bilateria is yet to be unequivocally identified. The hair cell receptor is therefore in all likelihood not older than any other vertebrate nicotinic receptor, be them of the muscle or neuronal subtypes. Nonetheless, α9α10 receptors are the most divergent nAChR in vertebrates. Future functional and molecular evolution studies will continue to shed light on the many peculiar features of α9α10 receptors and continue to contribute insight into the evolutionary history of nAChRs and that of the efferent modulation of mechanosensation.

## Author Contributions

All authors wrote the review and approved the submitted version.

## Conflict of Interest

The authors declare that the research was conducted in the absence of any commercial or financial relationships that could be construed as a potential conflict of interest.

## Publisher’s Note

All claims expressed in this article are solely those of the authors and do not necessarily represent those of their affiliated organizations, or those of the publisher, the editors and the reviewers. Any product that may be evaluated in this article, or claim that may be made by its manufacturer, is not guaranteed or endorsed by the publisher.
